# Use of Sodium–Glucose Cotransporter 2 Inhibitors in Hospitalized Patients

**DOI:** 10.1016/j.jacadv.2024.101024

**Published:** 2024-06-04

**Authors:** Ozan Unlu, Ankeet S. Bhatt, Alexander J. Blood

**Affiliations:** a Accelerator for Clinical Transformation, Brigham and Women’s Hospital, Boston, Massachusetts, USA; b Division of Cardiovascular Medicine, Brigham and Women’s Hospital, Boston, Massachusetts, USA; c Department of Biomedical Informatics, Harvard Medical School, Boston, Massachusetts, USA; d Harvard Medical School, Boston, Massachusetts, USA; e Department of Cardiology and Division of Research, Kaiser Permanente San Francisco Medical Center, San Francisco, California, USA; f Department of Medicine, Stanford University School of Medicine, Palo Alto, California, USA

**Keywords:** guideline-directed medical therapy, in-hospital initiation, Sodium–glucose cotransporter 2 inhibitors

## Abstract

Sodium–glucose cotransporter 2 inhibitors (SGLT2i) have noted benefits in the treatment of type 2 diabetes, cardiovascular disease, heart failure, and chronic kidney disease. Despite these benefits, the adoption of SGLT2i in clinical practice has been slow. Early initiation of SGLT2i during hospitalization has been proposed to address this gap for 2 important reasons: 1) it provides early clinical benefit in multiple disease states; and 2) hospitalization presents an opportunity for medication optimization and patient education, thereby overcoming clinical inertia. Challenges in SGLT2i adoption necessitate innovative strategies for integration into clinical practice. Ongoing trials and novel care delivery models are anticipated to further elucidate effective strategies for SGLT2i implementation and adherence. This review synthesizes the accrued evidence of SGLT2i across various chronic diseases. It emphasizes the rationale for early in-hospital initiation and discusses barriers and potential solutions for widespread implementation of SGLT2i in hospitalized patients.

## Highlights

• SGLT2 inhibitors are effective in managing type 2 diabetes, cardiovascular diseases, heart failure, and chronic kidney disease.
• Initiating SGLT2 inhibitors early during a hospital stay offers a strategic approach to optimize patient care and overcome barriers to initiation.
• Advancements in care delivery methods will focus on addressing barriers to adoption and integrating innovative care models for wider implementation.

Sodium–glucose cotransporter 2 inhibitors (SGLT2i) prevent glucose reabsorption in the proximal renal tubule and emerged as effective treatments in modestly improving glycemic control for type 2 diabetes (T2D).^1^ More recently, these therapies have been shown to improve clinical outcomes across a much wider range of conditions including cardiovascular (CV) disease and heart failure (HF) across the spectrum of left ventricular ejection fraction (LVEF) with or without T2D and chronic kidney disease (CKD).

Despite these benefits, the uptake of SGLT2i has been slow leading to significant gaps in care for patients who would benefit.^2^ Early initiation of SGLT2i during a hospital stay has been proposed as an implementation strategy to help close this care gap and to fully realize the potential population level benefits of the medication seen after a hospitalization.^3^ In hospital, implementation affords many potential benefits, including disease and symptom monitoring, availability of hemodynamic and lab assessments, and time for reinforming, medication education, and provider-patient dialog. To capitalize on this opportunity, it is paramount to educate clinicians and develop and evaluate strategies for in-hospital initiation of SGLT2i and subsequent maintenance after discharge. In this review, we aim to present the evidence for the effectiveness and safety of SGLT2i in the treatment of a variety of chronic diseases, establish the rationale for early, in-hospital initiation, and discuss potential barriers and solutions for widespread in-hospital implementation of SGLT2i.

## SGLT2i in the treatment and prevention of chronic diseases

SGLT2i have demonstrated improved outcomes for patients with HF across the spectrum of LVEF in a number of pivotal clinical trials. For patients with HF with reduced ejection fraction (HFrEF), EMPEROR-reduced,^4^ DAPA-HF,^5^ SOLOIST-WHF,^6^ and DEFINE^7^ provided the evidence that ultimately led to inclusion of SGLT2i as a Class I recommendation for HFrEF in the contemporary HF guidelines.^4^, ^5^, ^6^, ^7^, ^8^, ^9^ Similar to those with HFrEF, pivotal trials for patients with HF with preserved ejection fraction (HFpEF) including EMPEROR-Preserved,^10^ DELIVER,^11^ PRESERVED-HF,^12^ and some participants from SOLOIST-WHF^6^ established the efficacy of SGLT2i.^6^^,^^10^, ^11^, ^12^, ^13^, ^14^ These studies provided the first evidence of benefit from the addition of HF therapy for patients with improved ejection fraction, adding to evidence surrounding initiation and continuation of guideline-directed medical therapy (GDMT).^15^

Several major trials established the efficacy of SGLT2i in patients with T2DM and established CV disease. These included the CANVAS Program,^16^ DECLARE-TIMI 58 trial,^17^ EMPA-REG OUTCOME,^18^ SCORED trial^19^ which not only established the CVD benefit of SGLT2i in patients with T2D but also laid the groundwork for further studies to illustrate their benefits in patients with HF and CKD. These trials not only established the CVD benefit of SGLT2i in patients with T2D but also laid the groundwork for further studies to illustrate their benefits in patients with HF and CKD.

Finally, 3 major trials evaluated the efficacy of SGLT2i in patients with CKD with and without T2DM including the CREDENCE trial^20^, DAPA-CKD trial ^21^, and the EMPA-Kidney trial.^22^ These trials demonstrated in patients with both reduced estimated glomerular filtration rate (eGFR) and albuminuria with SGLT2i, leading to reductions in kidney disease progression and kidney and CV-associated death.

The aforementioned pivotal trials and their key components are listed in Table 1. On the basis of these trials, the US Food and Drug Administration approved the following: 1) in 2016, empagliflozin to reduce the risk of CV death in adults with T2D and established CV disease; 2) in September 2019, canagliflozin to treat diabetic kidney disease; 3) in May 2020, dapagliflozin for HFrEF; 4) in May 2021, dapagliflozin to treat patients with CKD at risk of disease progression, regardless of diabetes and albuminuria status; and 5) in September 2022, empagliflozin for HFpEF, and September 2023, empagliflozin for CKD without DM or HF.

**Table 1:** Pivotal Trials for Sodium–Glucose Cotransporter 2 Inhibitor

Trial Name	Patient Population	Drug Tested	Comparison	Primary Endpoints	Key Findings
HFrEF					
EMPEROR-Reduced	HFrEF; NYHA functional class II, III, or IV	Empagliflozin	Placebo	Composite of CV death or hospitalization for HF	Empagliflozin significantly reduced the risk of CV death or hospitalization for HF in patients with HFrEF compared to placebo, irrespective of the presence or absence of diabetes.
DAPA-HF	HFrEF; NYHA functional class II, III, or IV	Dapagliflozin	Placebo	Composite of worsening HF or death from CV causes	Dapagliflozin reduced the risk of worsening HF or death from CV causes among patients with HFrEF compared to placebo, regardless of diabetes status. In patients without a previous history of HF hospitalization, dapagliflozin decreased the relative risk of the primary outcome by 16%. For those who had been hospitalized for HF more than a year before joining the study, the reduction was 27%, and for those who had an HF hospitalization within the 12 mo prior to entering the trial, the risk reduction was 36%.
DEFINE	HFrEF; NYHA functional class II or III, eGFR ≥30 mL/min/1.73 m^2^, elevated natriuretic peptide	Dapagliflozin	Placebo	Composite of improvement in KCCQ overall summary score or NT-proBNP level	Dapagliflozin improved the composite outcome of Kansas City Cardiomyopathy Questionnaire (KCCQ) overall summary score or NT-proBNP level in patients with HFrEF.
SOLOIST-WHF	T2D and worsening HF across the EF spectrum	Sotagliflozin	Placebo	Composite of total number of deaths from CV causes, hospitalizations, and urgent visits for HF	Sotagliflozin led to a lower rate of primary-endpoint events than placebo in patients with T2D and worsening HF across the EF spectrum.
HFpEF					
EMPEROR-Preserved	HFpEF; NYHA functional class II, III, or IV	Empagliflozin	Placebo	Composite of CV death or hospitalization for HF in patients with HFpEF	Compared to placebo, empagliflozin reduced the risk of CV death or hospitalization for HF in patients with HFpEF, regardless of the diabetes status.
DELIVER	HFpEF or mildly reduced EF; with or without diabetes, with signs and symptoms of HF, elevated natriuretic peptide	Dapagliflozin	-	Composite of worsening HF and CV death; improvement in symptom burden measured by KCCQ total symptom score	Dapagliflozin led to a lower rate of composite endpoint of worsening HF and CV death and improved symptom burden determined by KCCQ total symptom score. Dapagliflozin reduced the risk of worsening HF or CV death by 22% in recently hospitalized patients and by 18% in nonhospitalized patients.
PRESERVED-HF	HFpEF; NYHA functional class II, III, or IV, elevated natriuretic peptide, loop diuretic use with HF hospitalization within the past 12 mo	Dapagliflozin	-	Improvement in health status, functional ability, and quality of life in patients with HFpEF	Dapagliflozin improved health status, functional ability, and quality of life in patients with HFpEF.
T2DM					
CANVAS Program	Patients with T2DM and high CV risk; 65.6% had a history of CV disease at baseline	Canagliflozin	Placebo	Composite of death from CV causes, nonfatal myocardial infarction, or nonfatal stroke	Canagliflozin reduced the risk of CV events in patients with T2D and high CV risk; however, it increased the risk of amputation. The primary outcome was a composite of death from CV causes, nonfatal myocardial infarction, or nonfatal stroke.
DECLARE-TIMI 58	Patients with T2DM and established ASCVD or multiple risk factors for ASCVD	Dapagliflozin	Placebo	Composite of CV death or hospitalization for HF; major adverse CV events (CV death, myocardial infarction, or ischemic stroke)	Dapagliflozin resulted in lower rates of CV death or hospitalization for HF than placebo among patients with T2D and ASCVD or multiple risk factors for ASCVD (HR: 0.83; 95% CI: 0.73-0.95; *P* = 0.005); however, it did not result in a lower rate of major adverse CV event defined as CV death, myocardial infarction, or ischemic stroke.
EMPA-REG OUTCOME	Patients with T2D and high CV risk	Empagliflozin	Standard care	Composite of CV death, nonfatal myocardial infarction, or nonfatal stroke	Empagliflozin, when added to standard care, reduced the risk of CV death, nonfatal myocardial infarction, or nonfatal stroke among patients with T2D and high CV risk.
SCORED	Patients with T2DM and CKD; eGFR of 25-60 ml/min/1.73 m^2^	Sotagliflozin	Placebo	Composite of death from CV causes, hospitalizations, and urgent visits for HF	Sotagliflozin led to a lower rate of death from CV causes and hospitalizations and urgent visits for HF compared to placebo in patients with T2D and CKD with an eGFR of 25-60 ml/min/1.73 m^2^.
CREDENCE	Patients with T2D and CKD; eGFR of 30 to 90 ml/min/1.73 m^2^ and UACR of 300 to 5000 mg/g	Canagliflozin	Placebo	Composite of ESKD, a doubling of the serum creatinine level, or death from kidney or CV causes	Canagliflozin significantly reduced the primary outcome (composite of ESKD, a doubling of the serum creatinine level, or death from kidney or CV causes) compared to placebo in patients with T2D and CKD with an eGFR of 30 to 90 ml/min/1.73 m^2^ and a UACR of 300 to 5,000 mg/g.
CKD					
DAPA-CKD	Patients with CKD; eGFR of 25 to 75 ml/min/1.73 m^2^ and UACR of 200 to 5,000 mg/g	Dapagliflozin	Placebo	Composite of sustained decline in the eGFR of at least 50%, ESKD, or death from kidney or CV causes	In the dapagliflozin group, the primary composite outcome (sustained decline in the eGFR of at least 50%, ESKD, or death from kidney or CV causes) was significantly reduced compared to the placebo group in patients with CKD and a UACR of 200 to 5,000 mg/g.
EMPA-Kidney	Patients with CKD; eGFR of 20 to 45 ml/min/1.73 m^2^ or 45 to 90 ml/min/1.73 m^2^ and UACR of ≥200 mg/g	Empagliflozin	Placebo	Composite of kidney disease progression or death from CV causes	The primary outcome (composite of kidney disease progression or death from CV causes) was significantly lower in the empagliflozin group than the placebo group, with consistent results in those with or without diabetes and across the range of eGFR studied, in patients with CKD and an eGFR of 20 mL/min/1.73 m^2^

CKD = chronic kidney disease; CV = cardiovascular; eGFR = estimated glomerular filtration rate; HF = heart failure; HFrEF = HF with reduced ejection fraction; T2D = type 2 diabetes.

## Initiation of SGLT2i in hospitalized patients

The initiation of SGLT2i in hospitalized patients has been a point of discussion on the basis of 2 important arguments: 1) potential benefits of early initiation of SGLT2i; and 2) hospitalizations providing an additional touch point for medication optimization and monitorization of potential adverse events (Central Illustration).

**Central Illustration:**
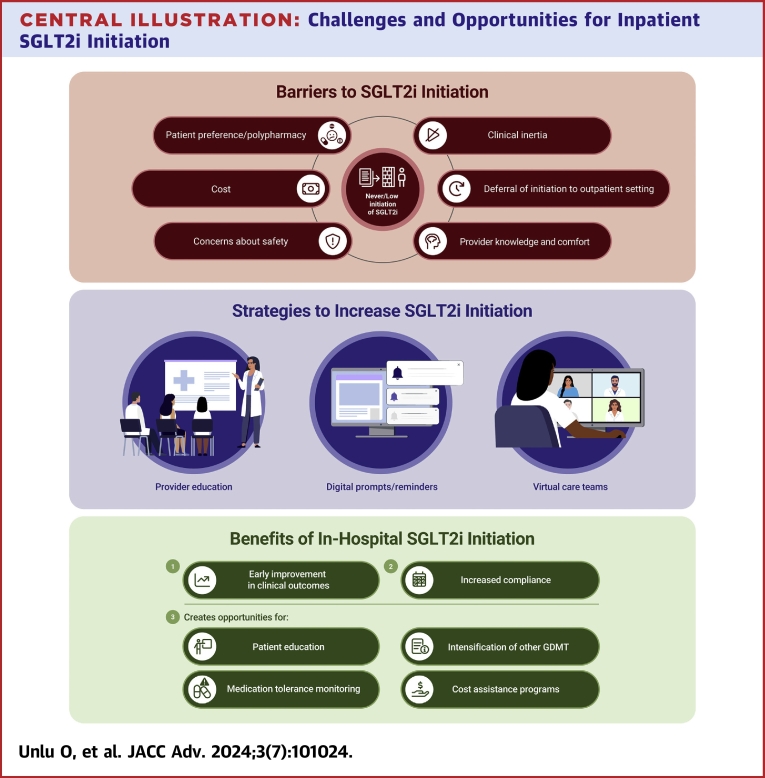
Challenges and Opportunities for Inpatient SGLT2i Initiation

### Benefit of early initiation of SGLT2i

The SOLOIST-WHF and EMPULSE trials have underscored the advantages of commencing SGLT2i treatment early. In the SOLOIST-WHF study which included patients hospitalized due to worsening HF, the administration of sotagliflozin, which is a both SGLT2 and 1 inhibitor, either immediately prior to or shortly following discharge, resulted in a significant decrease in death from CV causes and hospitalizations and urgent visits for HF (HR 0.67; 95% CI 0.52-0.85; *P* < 0.001).^6^

Similarly, the EMPULSE trial included patients hospitalized with a diagnosis of acute de novo or decompensated chronic HF and initiated empagliflozin when the patients were clinically stable in the hospital. The primary outcome was clinical benefit that was assessed using a win ratio and was defined as a hierarchical composite of death from any cause, number of HF events and time to first HF event, or a 5 point or greater change in KCCQ Total Symptoms Score at 90 days. Patients in the empagliflozin group had a higher rate of clinical benefit than the placebo group (stratified win ratio: 1.36; 95% CI: 1.09-1.68; *P* = 0.0054) regardless of the ejection fraction, acute vs chronic HF, or the presence of diabetes.^23^

In addition, secondary analyses of DAPA-HF, EMPEROR-Reduced, EMPEROR-Preserved, and DELIVER were conducted to investigate time to clinical benefit of SGLT2i in HF across the LVEF spectrum. DAPA-HF showed that the composite of CV death or worsening HF was lower with dapagliflozin. The statistical significance of this benefit was sustained by 28 days after randomization (HR at 28 days: 0.51 [95% CI: 0.28-0.94]; *P* = 0.03). The upper confidence boundary of the HR remained below unity for the remainder of the trial. A similar pattern of early and consistent benefit was observed for the individual components of the primary efficacy outcome (worsening HF: HR at 28 days 0.48, 95% CI: 0.23-0.94; CV death: HR at 28 days 0.87, 95% CI: 0.31-2.41). EMPEROR Reduced trial included patients with HFrEF to investigate the effect of empagliflozin compared to placebo for a primary endpoint of CV death or hospitalization for HF. The study found that empagliflozin significantly reduced the primary composite endpoint in empagliflozin group compared to placebo (HR: 0.75; 95% CI: 0.65-0.86; *P* < 0.001). The benefit of empagliflozin on this endpoint first reached statistical significance at 12 days after randomization, and statistical significance was sustained from day 34 onwards. Similarly, The EMPEROR-Preserved trial studied the effects of empagliflozin in patients with HFpEF and found that empagliflozin reduced the composite outcome of CV death or HF hospitalization compared to placebo. These effects were seen early and consistently. Statistical significance for separation between the empagliflozin and the placebo arms occurred by day 18 for time to CV death or HF hospitalization (HR at 18 days 0.41, 95% CI: 0.17-0.99), after which this benefit was sustained with the boundary of the upper CI below unity for the rest of the trial period. Finally, The DELIVER trial showed that dapagliflozin reduced CV death or worsening HF events in patients with HF with mildly reduced or preserved ejection fraction. The time to first nominal statistical significance for the primary endpoint was 13 days (HR: 0.45; 95% CI: 0.20-0.99; *P* = 0.046), and significance was sustained from day 15 onwards. First and sustained statistical significance was reached for worsening HF events (HR: 0.45; 95% CI: 0.21-0.96; *P* = 0.04) by day 16 after randomization. Significant benefits for the primary endpoint and worsening HF events were sustained at 30 days, 90 days, 6 months, 1 year, 2 years, and final follow-up (primary endpoint: HR: 0.82; 95% CI: 0.73-0.92; worsening HF events: HR: 0.79; 95% CI: 0.69-0.91).

### Using hospitalization as an opportunity for medication optimization

Clinical inertia is one of the most important barriers for initiation of GDMT in HF with many potential contributing physician, patient, and system factors. The fear of medication adverse events is one of the major reasons for physician and patient clinical inertia.^24^ Hospitalizations are additional touch points in the care of some patients with HF and they offer a unique opportunity to overcome inertia by allowing close monitoring of adverse events after initiation or up-titration of medications. In addition, it also gives the clinical team a chance to educate the patient about their disease and potential treatments, as well as address potential issues around cost before discharge.

During hospitalization, there is a unique opportunity to educate patients about HF, its management, and the importance of adhering to the medication regimen. Studies have shown that effective communication about reasons for medication prescriptions and potential adverse drug events is critical and can improve adherence to therapies. Therefore, hospitalizations can provide the time for various members of the clinical team to educate the patients and reinforce the importance of HF therapies.^25^ In addition, hospitalizations provide an opportunity for a monitored setting to observe tolerability of prescribed HF therapies where any adverse events can be promptly addressed. It might also prevent reactionary and incorrect discontinuation of therapies based on findings such as an expected transient change in renal function, described as eGFR dip. The initial decline in eGFR after initiation of dapagliflozin in patients with HFrEF was found to be relatively small and was associated with better clinical outcomes compared to a similar decline on placebo.^26^

## Potential barriers and solutions for initiation of SGLT2i in hospitalized patients

Clinical inertia or deferral to outpatient care can pose significant barriers to the initiation of SGLT2i in hospitalized patients. This is often due to perceived lower clinical risk or reluctance to initiate a new therapy, especially among patients who appear clinically stable with relatively mild symptoms.^24^ However, as discussed above, data from DAPA-HF, EMPEROR-Reduced, EMPEROR-Preserved, and DELIVER trials underscored the rapid clinical benefits observed with the SGLT2i, highlighting key opportunities for the early identification and prompt management of this patient population. Furthermore, in multiple observational studies, deferral of initiation of other GDMT has been shown to be associated with never initiation of GDMT.^27^, ^28^, ^29^ When the GDMT is started before hospital discharge, the compliance with GDMT posthospitalization and the chance of target-dose achievement in the short- and longer-term follow-up increases significantly.^29^ In the IMPACT-HF trial, at 60 days follow-up, patients started on carvedilol predischarge had higher use of beta-blockers than those who did not (91.2% vs 73.4%, *P* < 0.0001). Multiple strategies have been investigated to increase the initiation of GDMT during hospitalization. One potential strategy is to create teams to rapidly identify hospitalized patients with HF who are not on optimal medical therapy and intervene to recommend GDMT optimization. IMPLEMENT- HF investigated if this strategy can be implemented by using a virtual multidisciplinary team of physicians and pharmacists and showed that the virtual care team–based strategy improved the net intensification of GDMTduring a hospitalization (44% vs 24% for net intensification, *P* = 0.002).^30^ Similarly, in a pilot-randomized trial that investigated the efficacy of a virtual HF intervention on GDMT optimization on patients with HFrEF admitted with any cause showed a mean improvement of 0.75 in optimal medical therapy score at discharge in the intervention group compared.

An emerging concern among clinicians is the discharge of patients on SGLT2i, without ensuring adequate follow-up. This apprehension stems from potential challenges patients may face in adhering to their treatment plan, recognizing and managing side effects, and accessing necessary healthcare services postdischarge. Effective communication and establishing a clear, accessible follow-up plan are paramount to address these concerns. Therefore, the most recent AHA/ACC/HFSA guidelines for the management of HF highlight the importance of comprehensive care management, including the use of multidisciplinary teams. These teams can facilitate GDMT and support for managing symptoms, thus potentially reducing rehospitalization rates and improving survival outcomes for patients with HF.^8^ In a meta-analysis that pooled the randomized clinical trials investigating the effectiveness of pharmacist-involved multidisciplinary teams for the management of HF, it found a notable decrease in the rates of hospitalizations due to HF (OR: 0.72, 95%CI 0.55-0.93, *P* = 0.01, I2 = 39%) and in hospitalizations for any cause (OR: 0.76, 95% CI: 0.60-0.96, *P* = 0.02, I2 = 52%), which can be attributed, in part, to improved adherence to medication regimens. Additionally, they observed significant enhancements in the understanding of HF among participants.^31^

Provider knowledge and comfort with the medication class are crucial for the successful initiation of SGLT2i in hospitalized patients. Therefore, recurring and iterative education of providers regarding benefits and potential adverse effects of SGLT2i is important. In PROMPT-HF trial, providers were given access to a customized order set that displayed all available medications within the classes not currently prescribed, listed alphabetically along with their indication.^32^ This was supplemented with a hyperlink to the study webpage that contained informational documents expanding on evidence-based medical therapy recommended by current guidelines for patients with HFrEF. This approach was found to be effective in increasing provider knowledge and comfort level with HFrEF guidelines. In addition to the potential expected effects, providers should also be educated about adjustments in diabetic and diuretic regimens when initiating SGLT2is. Notably, in the pivotal trials, hypoglycemic events were rare and occurred at similar rates in those without diabetes.^4^^,^^5^ Nonetheless, combining SGLT2is with insulin or agents that stimulate insulin production (like glinides and sulfonylureas) could elevate hypoglycemia risk; therefore, it has been recommended to lower the dosage of sulfonylureas or glinides by half or reduce basal insulin by 20% upon initiating SGLT2i treatment, particularly if the patient's HbA1C is within the normal range or if they have a history of hypoglycemia.^33^^,^^34^ Similarly, studies investigated diuretic effects of SGLT2i, and its interplay with loop diuretics showed that it was effective across all spectrums of loop diuretic doses, and the diuretic dose did not change in most patients during follow-up and the change were similar to the placebo group.^35^ Therefore, provider education on the generally minimal to no adjustments required for diuretics could help reduce concerns related to monitoring and following up with patients.

A particular focus should be given to education about the expected effects of SGTL2i on eGFR. Concerns for a decline in eGFR are common when initiating SGLT2i therapy and can be a barrier for initiation or inappropriate discontinuation of SGLT2i. Importantly, a secondary analysis of the DAPA-HF trial showed that the mean reduction in eGFR after dapagliflozin was 4.2 ml/min/1.73 m^2^ compared to 1.1 ml/min/1.73 m^2^ in the placebo group. However, this initial decline was not associated with higher rates of long-term worsening kidney function or other adverse events. In fact, those with more than 10% eGFR decline in the dapagliflozin group had a lower rate of primary outcome which was a composite of worsening HF or CV death,^26^ suggesting this is likely to be a pharmcodynamic effect of the therapy rather than true acute kidney injury.

The safety data from existing clinical trials for SGLT2i showed that the number of safety events were low and consistent across trials. In the SOLOIST-WHF trial, sotagliflozin was administered to patients with diabetes who had recently been hospitalized for worsening HF. Diarrhea was more common with sotagliflozin than with placebo (6.1% vs 3.4%), as was severe hypoglycemia (1.5% vs 0.3%).^6^ However, the percentage of patients with hypotension was similar in the sotagliflozin group and the placebo group (6.0% and 4.6%, respectively), as was the percentage with acute kidney injury (4.1% and 4.4%, respectively). In the EMPULSE trial, empagliflozin was initiated in patients hospitalized for acute HF, regardless of LVEF. There were no significant differences of safety events between the empagliflozin group and the placebo group for volume depletion (12.7% vs 10.2%, respectively), hypoglycemia (1.9%, 1.5%, respectively), acute renal failure (7.7% vs 12.1%, respectively), and urinary tract infection (4.2% vs 6.4%, respectively).^23^ Overall, serious adverse events were reported in 32.3% of the empagliflozin-treated patients and 43.6% of the placebo-treated patients. These findings suggest that SGLT2i can be safely initiated in hospitalized patients, providing significant clinical benefits in the months following treatment initiation.

The cost and lack of cost transparency of SGLT2i is another significant consideration in their use. While the specific cost of these medications can vary based on factors such as location, insurance coverage, and specific medication, they are generally more expensive than some other classes of GDMT given that they have been added to the armamentarium of HF therapies. However, they can provide significant health benefits in patients with multiple comorbidities including T2D, CAD, CKD, and HF. Therefore, the trade-offs between health benefits of SGLT2i and increased costs should be carefully examined to justify their clinical use. The cost-effectiveness of treatments for HF, including SGLT2is, is a complex issue with multiple perspectives. The value of treatment is often framed within a willingness-to-pay model, which can vary significantly among stakeholders such as patients, healthcare providers, and insurance companies. Cost per quality-adjusted life year (QALY) is a common measure of value, with <$50,000 per QALY is considered high value and ≥$150,000 per QALY is considered low value.^36^ However, perspectives on what constitutes a cost-effective intervention can differ. For example, the American College of Cardiology and American Heart Association consider a health intervention that exceeds 3 times the GDP per capita per QALY as not cost-effective.^37^ From the patient's perspective, the concept of value also includes out-of-pocket costs and insurance premiums. The cost differences between HF treatments can significantly impact a patient's willingness to pay for a treatment and affect patients' adherence to treatment regimens.^38^ Therefore, the cost of SGLT2is is a crucial factor in their adoption and use, and it represents a significant barrier that needs to be addressed to improve patient outcomes. Importantly, in a cost-effectiveness analysis based on a hypothetical cohort with patients similar to DAPA-HF trial cohort showed that dapagliflozin had an incremental cost-effectiveness ratio of $68,300 per QALY gained (95% UI: $54,600-$117, 600 per QALY gained) which makes it cost-effective at current US prices.^39^ However, recent analyses from Medicare data underscore the significant financial burden these medications can impose on patients. In 2020, Medicare plans often required tier 3 cost-sharing for SGLT2i, with median annual out-of-pocket (OOP) costs for quadruple therapy, including SGLT2i, reaching $2,217.^40^ This is in stark contrast to a fully generic regimen, excluding SGLT2i/ARNIs, which had a median annual OOP cost of merely $3.^40^ Such high OOP costs likely render SGLT2i therapies unaffordable for many Medicare beneficiaries, despite their proven efficacy and the guidelines recommending their use in HFrEF. Moreover, the restriction of coverage through high-tier cost-sharing and the complexities around insurance policies further complicate patient access to these life-saving medications, highlighting a critical need for policy reforms aimed at reducing medication costs and improving transparency in drug pricing.^41^ Only through concerted efforts to address these cost barriers can we ensure broader patient access to SGLT2i, thereby leveraging their full potential in improving outcomes for patients with HF and other comorbid conditions.

Finally, patients with indications to use SGLT2i often have multiple comorbidities requiring several medications, which can lead to complex medication regimens. This complexity can lead to medication nonadherence, adverse drug reactions, and decreased quality of life which in turn can play a role in patients’ decisions to use SGLT2i. As an example, 1 observational study found that 84% of patients with HF used 5 or more medications at hospital admission and 42% used 10 or more medications.^42^ In addition, patients who have non-GDMT polypharmacy were less likely to achieve optimization of GDMT on follow-up.^43^ Therefore, careful review of patients’ medications should be performed for each patient and non-GDMT medications that pose harm or minimal benefit should be deprescribed. Hospitalizations provide a great opportunity to achieve this and monitor for potential adverse events following multiple medication changes. Furthermore, SGLT2i has been shown to reduce the risk of hyperkalemia, potentially enabling the use of renin aldosterone angiotensin inhibitors and mineralocorticoid receptor antagonists.^44^

We are on the verge of digital transformation in healthcare and innovative health delivery strategies that takes advantage of rapidly emerging technologies, which has the potential to address economic challenges in the treatment of HF. Remote patient encounters, telehealth, and mobile health are some of the platforms paving the way for an expansion of virtual care. These platforms are being used to gather health data, share it with healthcare providers, and empower patients for self-management of chronic diseases which can increase quality, reduce costs, and improve the coordination of care.^38^

## Ongoing trials and future directions

Several trials are currently underway to further investigate the benefits of SGLT2i in various patient populations and settings including EMPACT-MI, DICTATE-AHF, DAPA-ACT HF TIMI 68, and the ertugliflozin in AHF trial (Table 2). Furthermore, several implementation studies are being conducted to investigate a variety of interventions to improve SGLT2i prescriptions or broader GDMT initiation (Table 3).

**Table 2:** Ongoing SGLT2i Randomized Clinical Trials

	EMPACT-MI	DICTATE-AHF	DAPA-ACT HF TIMI-68	Ertugliflozin in AHF
Investigational treatment/control	Empagliflozin 10 mg/placebo	Dapagliflozin 10 mg plus diuretic therapy/diuretic therapy	Dapagliflozin 10 mg/placebo	Ertugliflozin/metolazone/placebo
Patient population	Patients hospitalized for acute myocardial infarction with high risk of HF defined as signs or symptoms of congestion or newly developed LVEF <45%	Patients with or without T2D hospitalized with acute decompensated HF	Patients who have been stabilized during hospitalization for acute HF	Acute and postacute hospitalized heart failure patients with or without diabetes
Primary endpoint	Time to first hospitalization for HF or all-cause death	Diuretic response measured by cumulative change in weight	Time to CV death or worsening heart failure	Change in urine sodium and total body water at days 1, 7, and 42
Key secondary endpoints	Total HHF or all-cause death Total nonelective CV hospitalizations or all-cause death Total nonelective all-cause hospitalizations or all-cause death Total hospitalizations for MI or all-cause death Time to CV death	Incidence of worsening HF Hospital readmission within 30 d	Time to CV death or rehospitalization for HF or urgent HF visit Time to CV death or rehospitalization for HF Time to rehospitalization for HF or urgent HF visit Hospital readmission within 30 d Time to CV death Time to all-cause death	

CV = cardiovascular; HF = heart failure; HHF = hospitalization for heart failure; LVEF = left ventricular ejection fraction; MI = myocardial infarction; T2D = type 2 diabetes.

**Table 3:** Ongoing Randomized Clinical Trials Investigating Care Delivery Methods to Initiate SGLT2i

Trial Name	ClinicalTrials.gov ID	Setting	Start Date	Estimated Completion Date	Intervention/Treatment	Population	Objective
COPILOT-HF: Cooperative Program for Implementation of Optimal Therapy in Heart Failure	NCT05734690	Mass General Brigham	May 2023	June 2025	Pharmacist and navigator driven remote care team	Patients with heart failure	Test 2 remote care strategies for optimizing GDMT prescription across the spectrum of LVEF: Education and remote management simultaneously vs education first followed by remote care
PROMPTHF-Inova: PRagmatic Trial Of Messaging to Providers About Treatment of Heart Failure at Inova	NCT05433220	Inova Health Care Services	August 2022	August 2024	Best Practice Advisory using Electronic Health Record	Patients with HFrEF	Test effectiveness of EHR best practice advisory to improve GDMT prescriptions for HF
Addressing Diffusion of Responsibility and Prescribing Burden to Improve Use of Diabetes Medications	NCT05463705	Brigham and Women's Hospital	May 2023	May 2024	Behavioral intervention to address diffusion of responsibility and simplification of prescribing	Primary care physicians caring for patients with type 2 diabetes and HbA1c >7.5% with a compelling indication for SGLT-2i or GLP-1RA	Test impact of addressing diffusion of responsibility on SGLT-2i and GLP-1RAs prescribing
INITIATE-HFrEF	NCT05989503	Universidade do Porto	August 2023	February 2025	Drug: Sacubitril-valsartan, Drug: SGLT2 inhibitor	Patients with HFrEF	Compare simultaneous vs sequential initiation of ARNi and SGLT2i for safety and efficacy
GREAT-HF Care	NCT05990296	Geisinger Clinic	August 2023	January 2025	Behavioral: Multiprong CDS with referral to pharmacist co-management, Behavioral: Multiprong CDS with GDMT order set, Behavioral: Focused education	HFrEF	Evaluate a multifaceted interdisciplinary intervention to improve GDMT use, reduce mortality, and future heart failure events in patients with HFrEF

GDMT = guideline-directed medical therapy; HF = heart failure; HFrEF = HF with reduced ejection fraction; LVEF = left ventricular ejection fraction; SGLT2i = sodium–glucose cotransporter 2 inhibitor.

In addition to possibly expanding the role of SGLT2i with the results of these trials, implementation research will be fundamental in accelerating the uptake and utilization of proven therapies and establishing innovative care delivery models such as PROMPT-HF and IMPLEMENT-HF. It is highly important to examine not only the efficacy of new care delivery models but also their impacts on costs and burden on patients and providers. Another approach to rapid initiation of SGLT2i has been to initiate and titrate the medications either right before or shortly after the hospitalization. STRONG-HF investigated such a strategy with initiation of 4 class GDMT at hospital discharge for patients with HFrEF followed by rapid up-titration posthospitalization.^45^ The study showed that the intensive strategy reduced the composite of HF readmission or all-cause death up to day 180 and did not increase serious adverse events. However, real-world implementation and scaling of such a program requires substantial resources and is difficult to sustain when the costs associated with HF management are already excessive. As a potential mitigation strategy, remote care strategies can be effective. A remote team-based strategy in a nonrandomized study using algorithms was shown to be a successful strategy for rapid GDMT initiation in patient with HFrEF.^46^ To further investigate this strategy in a larger patient population with all HF, the COPILOT-HF trial (NCT05734690) was designed. COPILOT-HF is a pragmatic randomized trial to determine if a remote clinic that implements a standardized, stepped-approach to medication optimization in patients with HF will achieve a higher rate of GDMT than a strategy of patient and provider education followed by remote HF clinic management.

## Conclusions

SGLT2i have shown significant promise in the treatment and prevention of chronic diseases such as HF, T2D, CAD, and CKD. The results of various clinical trials have demonstrated the efficacy and safety of SGLT2i in these patient populations. Despite the evidence, utilization of SGLT2i remains low and hospitalizations provide a potential opportunity to improve prescription rates. To overcome the potential barriers to initiation of SGLT2i in hospitalized patients, such as clinical inertia, provider knowledge, cost, and patient preference, innovative strategies need to be developed. Ongoing trials to expand the indications of SGLT2i as well as novel care delivery models are expected to provide further insights into the optimal use of SGLT2i in various clinical settings and appropriate integration into practice.

## Funding support and author disclosures

This work was supported by Boehringer Ingelheim Pharmaceuticals, Inc. (BIPI) and Lilly USA, LLC. The authors meet criteria for authorship as recommended by the International Committee of Medical Journal Editors (ICMJE). The authors received no payment related to the development of the manuscript. Graphical support was provided by Envision Pharma Group, contracted and funded by Boehringer Ingelheim Pharmaceuticals, Inc (BIPI) and Lilly USA, LLC. BIPI and Lilly were given the opportunity to review the manuscript for medical and scientific accuracy as well as intellectual property considerations. Dr Unlu has received funding from the 10.13039/100000050National Heart Lung and Blood Institute under award number T32HL007604. Dr Bhatt has received grant support to his institution from 10.13039/100000002National Institutes of Health/10.13039/100000050National Heart, Lung, and Blood Institute, 10.13039/100000002National Institutes of Health/10.13039/100000049National Institute on Aging, 10.13039/100005485American College of Cardiology Foundation, and the 10.13039/100000030Centers for Disease Control and Prevention and has received consulting fees from Sanofi Pasteur, Novo Nordisk, and the Kinetix Group. Dr Blood has received grant support from Novo Nordisk, 10.13039/100001003Boehringer Ingelheim, GE Healthcare, 10.13039/100004312Eli Lilly; consulting fees from Walgreens Health, Color Health, Flare Capital, Arsenal Capital Partners, Novo Nordisk, Milestone Pharmaceuticals; and has equity holdings in Knownwell; and has received grant support from National Institutes of Health/ National Heart, Lung, and Blood Institute and under Equity “Signum Technologies and Porter Health”.
